# Dupilumab-Induced Psoriasiform Eruption in an 83-Year-Old Patient

**DOI:** 10.7759/cureus.109041

**Published:** 2026-05-17

**Authors:** Vy X Pham, Erik Gilbertson

**Affiliations:** 1 Dermatology, UC San Diego, La Jolla, USA; 2 Dermatology, Scripps Clinic, La Mesa, USA

**Keywords:** atopic dermatitis, dermatology, drug-induced psoriasis, dupilumab, geriatrics, secukinumab

## Abstract

This case report describes an 83-year-old female who developed severe psoriasiform lesions suspected to be induced by dupilumab. The patient initially presented with eczematous papules affecting her back, arms, torso, and scalp, and a biopsy confirmed a subacute eczematous process. Treatment with topical, intralesional, and oral steroids failed to show significant improvement. Given the recalcitrant nature of her dermatitis, she was started on dupilumab injections, which initially offered temporary resolution and regression of her existing lesions. However, two weeks after initiating dupilumab treatment, the patient’s lesions relapsed and progressively worsened. A repeat biopsy at this time showed psoriasiform epidermal hyperplasia, and dupilumab treatment was stopped. The patient was ultimately transitioned to secukinumab, an IL-17 inhibitor, which resolved her flare.

## Introduction

Atopic dermatitis (AD) is a chronic inflammatory skin condition that often initially presents in childhood and is characterized by itchy, dry, erythematous, and lichenified skin lesions. The persistence of this condition into adulthood is not uncommon, with a high lifetime prevalence of 34.1% [1]. There are fewer documented cases of adult-onset AD in the absence of any childhood precipitating factors. For these patients, other diagnoses, such as contact dermatitis, malignancy, drug reaction, or infection, should be ruled out first before considering a diagnosis of AD. Regardless of age, standard management of this condition involves the use of topical corticosteroids and in refractory cases, targeted biologic treatment. Several key inflammatory markers have been identified in the pathogenesis of AD, such as IL-4 and IL-13, which help mediate adaptive immunity through promoting immune cell differentiation and activating other immune cells. The role of IL-4 and IL-13 in AD is demonstrated by the efficacy of dupilumab treatment, which specifically targets these immune mediators [2].

While the safety profile of dupilumab has been well-documented in the literature, several case reports and clinical trials have shown rare complications, which include debilitating psoriasiform eruption. This rare drug reaction is thought to be mediated by the inhibition of IL-4, which normally regulates the production of IL-17, a key mediator driving psoriasis [3]. However, most of the clinical trials and case reports that document this reaction have excluded patients over 60 years old, and a systematic review of these trials showed that only 4% of participants enrolled in dupilumab trials were aged 65 years or older [4,5]. This is significant, as the immune reactivity of a geriatric patient may vastly differ from that of a younger patient, and the currently available safety data on emerging biologic therapies may not be generalizable to all age groups. Therefore, this case of dupilumab-associated psoriasis in an 83-year-old patient offers new data to address this gap in the literature, especially for dermatologists, and navigate treatment resistance in complex or refractory dermatitis cases in elderly patients.

Consent for publication of this case report and any accompanying images was obtained from the patient.

## Case presentation

Initial presentation

An 83-year-old female presented with subacute flaring of pruritic, erythematous papules with no overlying scale, involving her trunk, flexural surfaces of her arms and legs, and bilateral inframammary folds. She had been using an over-the-counter absorbent powder upon symptom onset, with minimal improvement in her lesions, and treatment with topical corticosteroids provided little relief. She denied any new medications, personal care products, or travel, and her medical and family history were negative for allergies and atopy. The patient also denied any personal or family history of psoriasis or psoriatic arthritis symptoms.

**Figure 1:**
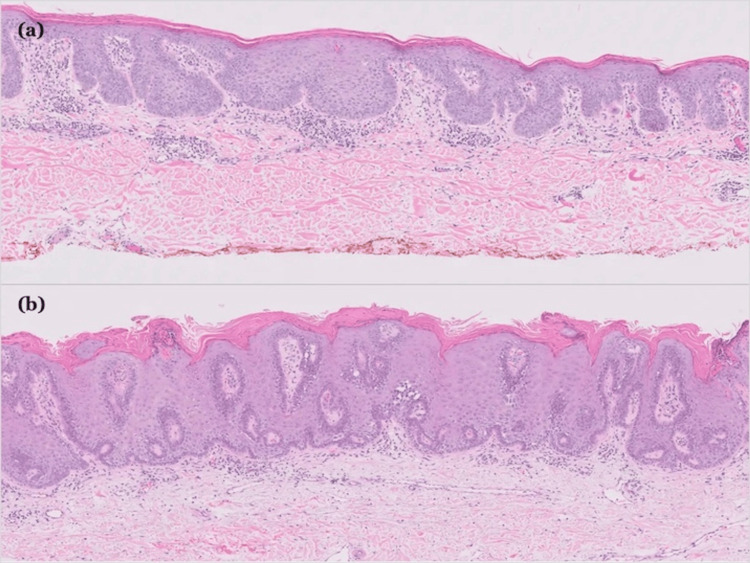
Skin biopsy (a) 10x skin biopsy using hematoxylin and eosin (H&E) staining showing spongiotic dermatitis with eosinophils before starting dupilumab. Superficial perivascular inflammatory infiltrate is also notable. (b) 10x skin biopsy using H&E staining showing mild, patchy parakeratosis with alternating orthohyperkeratosis and a predominantly preserved granular cell layer overlying irregular epidermal acanthosis and spongiosis consistent with psoriasiform changes after starting dupilumab. Compared to (a), rete ridges are notably elongated and acanthosis is regular, which is a characteristic finding of psoriatic processes.

Although serum testing was negative for eosinophilia, a biopsy of the lesion showed spongiotic dermatitis with eosinophils (Figure 1a). Multiple differential diagnoses were considered, including AD, paraneoplastic processes, drug eruption, contact dermatitis, cutaneous T-cell lymphoma (CTCL), infection, among other less likely considerations. Paraneoplastic and malignant processes, such as CTCL, were considered, given the patient's age. However, given negative malignancy screening (e.g., mammography and colonoscopy), the absence of other constitutional symptoms, normal lactic acid dehydrogenase levels, and lack of histologic evidence of CTCL, suspicion of an underlying cancer was low. Drug eruption and contact dermatitis were ruled out, given the negative patient history of new changes in medications or exposures to allergens consistent with lesion distribution. Infection was less likely given negative evidence of bacterial or fungal organisms on pathology, and the absence of leukocytosis or fever. Before presentation to the dermatology clinic, the patient had also completed an empiric 10-day course of gentamicin, with no improvement. Direct immunofluorescence studies were negative, arguing against an autoimmune bullous disorder. Other considerations included inverse psoriasis given involvement of the inframammary folds, which could have resulted from koebnerization of the multiple seborrheic keratoses scattered over the areas of involvement. However, given flexural involvement and the distribution of lesions being atypical for inverse psoriasis outside of the inframammary folds, a diagnosis of AD was ultimately favored. 

The patient received two intramuscular triamcinolone acetonide injections and fluocinolone, with no improvement. Topical betamethasone ointment and high doses (40 mg) of oral prednisone provided mild resolution, but tapering of steroid doses resulted in recurrent flaring of the lesions and pruritus.

Treatment

Given limited clinical improvement despite these therapies, the patient was started on weekly dupilumab injections. Initially, partial resolution was noted following a 600 mg injection with markedly decreased erythema and no new lesions. However, the patient was noted to have worsening erythema two weeks after her initial injection. Concerned that her eczema had progressed, the patient was given a second 300 mg injection of dupilumab but returned to clinic two days after with rapid exacerbation of well-defined, scaly plaques on a diffuse background of erythema and progressively worsening pruritus over her back, arms, torso, and scalp.

A repeat biopsy showed mild, patchy parakeratosis with alternating orthohyperkeratosis and a predominantly preserved granular cell layer overlying irregular epidermal acanthosis and spongiosis (Figure 1b). Rare eosinophils were noted, and staining for fungal organisms was negative. Direct immunofluorescence studies were negative. These findings were most consistent with a diagnosis of a psoriasiform spongiotic dermatitis with eosinophils. Given the worsening flares and new histopathologic findings following dupilumab administration, a psoriasiform drug eruption secondary to dupilumab administration was suspected. Dupilumab was discontinued, and upadacitinib, a Janus kinase-1 inhibitor, was started, given the preference for a medication with a lower loading dose (45 mg); however, this was soon discontinued after a single administration due to the unfavorable side effects of severe dizziness and gastrointestinal upset.

**Figure 2:**
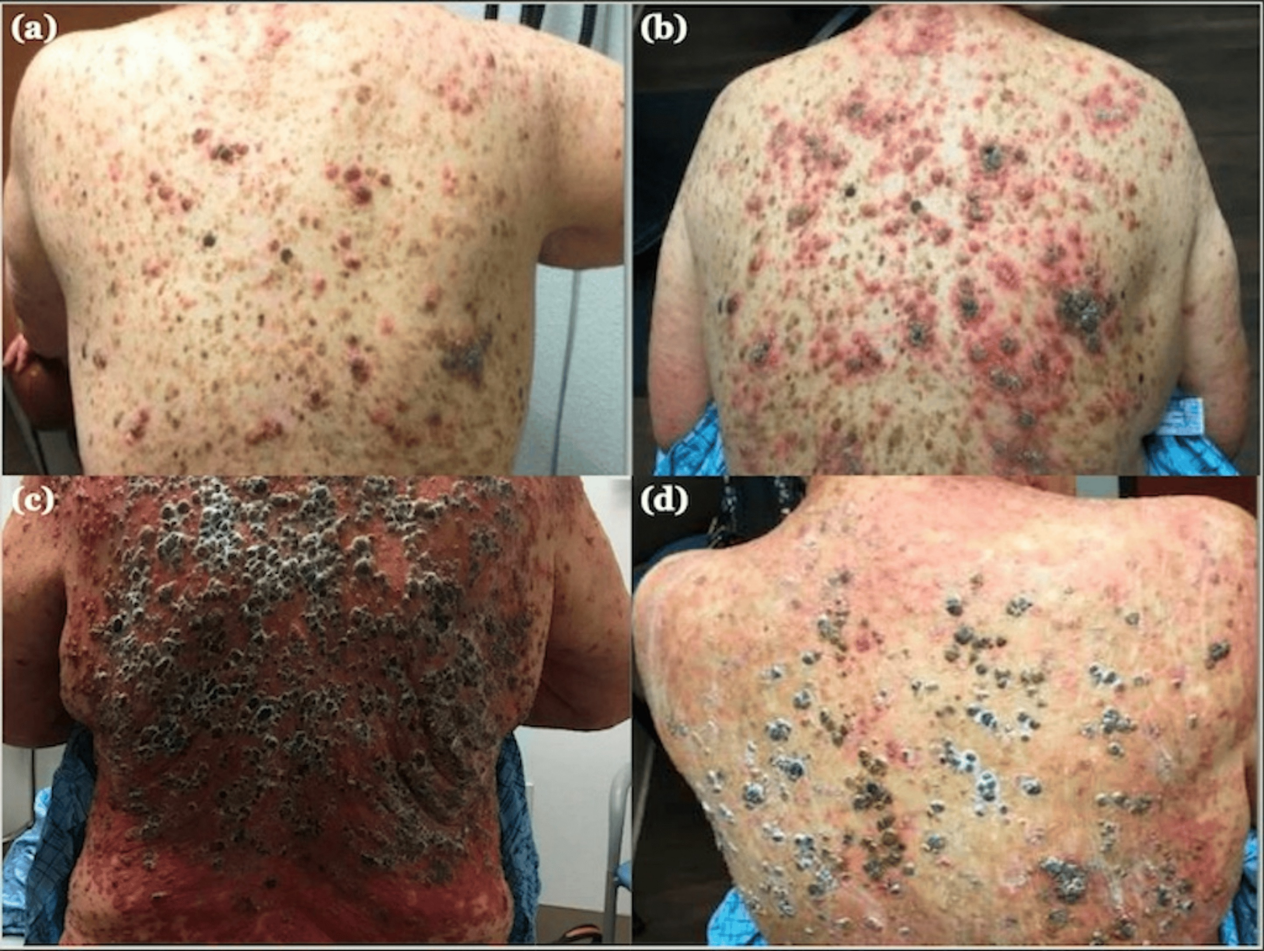
Progression of cutaneous lesions (a) Initial improvement with 300 mg dupilumab injection (b) Worsening erythema two weeks after initial injection (c) Rapid exacerbation of erythema, scaling, crusting anatomical involvement, and plaque formation two days after 600 mg dupilumab injection (d) Improvement of cutaneous eruption after two months of secukinumab with significantly reduced erythema, scaling, and overall number of plaques

Given the patient's age, other treatments that required closer follow-up, such as methotrexate, cyclosporine, or phototherapy, were considered less favorable, and the patient was then started on weekly 150 mg secukinumab injections instead. Within a week, this treatment resulted in significant improvement in pruritus and erythema. The patient was continued on a weekly 150 mg injection of secukinumab for five weeks, then transitioned to 300 mg once a month, with consistent improvement (Figures 2a-2d). 

Follow-up

Before starting this treatment, it was estimated that 50% of the patient’s body surface area was affected. After two months of secukinumab, the patient now presents with only 10% involvement of her total body surface area and significant improvement in pruritus and pain. Given the timeline of exacerbation following dupilumab administration, improvement after discontinuation and initiation of an IL-17 antagonist, and psoriasis as a documented possible adverse drug reaction to dupilumab, the diagnosis favored was a psoriasiform drug eruption secondary to dupilumab, as opposed to other considerations such as true psoriasis or psoriasiform eczema. Rebound psoriasis resulting from oral prednisone discontinuation was considered; however, the patient had been gradually tapered off of prednisone several weeks before starting dupilumab without psoriatic flaring, making this diagnosis less likely. 

## Discussion

Given the acuity of symptoms following dupilumab administration and resolution with IL-17 targeted therapy, our case is most consistent with drug-induced psoriasis secondary to dupilumab. Dupilumab is often used for refractory AD but has a documented 1.8%-3.3% risk of drug-associated psoriasis [6]. Lin et al. demonstrated the increased risk of de novo psoriasis in individuals with AD treated with dupilumab, with an incidence rate of 2.86% compared with 1.79% in the control cohort [7].

The mechanism mediating dupilumab-induced psoriasis continues to be explored. Su and Zeng propose that this is due to a T-helper 17 and T-helper 2 (Th17 and Th2) polarization spectrum, in which blocking Th2 cell pathways, which mediate AD pathogenesis, shift this spectrum toward Th1/Th17 pathways, driving psoriatic lesions to form [3]. Furthermore, Napolitano et al. demonstrated increased levels of IL-23A cytokines in de novo psoriatic lesions in patients with AD, supporting increased activity of the signaling pathway, which drives IL-17 production [8]. This case most likely aligns with this proposed shift, as demonstrated by the flare of psoriatic lesions following the inhibition of Th2 with dupilumab, and subsequent resolution with secukinumab, a selective IL-17A inhibitor, in our patient [9]. 

However, additional considerations should be taken when considering patients aged 60 years and older, with AD in this demographic being recognized as a separate clinical variant from that of younger patients presenting with AD [10]. Recent studies argue that over several decades of mediating stressors, the immune system of geriatric patients is at a higher likelihood of becoming dysregulated -- a phenomenon that has been labeled as "immunosenescence." This is supported by data showing chronically elevated inflammatory markers among these individuals, which represent the imbalance between immune system activation and regulation with age [11]. While it is unclear whether such dysregulation impacts the susceptibility of these patients to conditions such as atopic dermatitis or drug-associated psoriasis, our case reflects a treatment approach that centers on minimizing adverse effects and treatment burden in elderly patients, given the available drug safety data and recommended dosing regimens. While our patient did not have chronic medical conditions and was not taking other medications at the time of presentation, the majority of patients in this population face complications with their treatment due to comorbidities and polypharmacy, requiring a balance between efficacy and safety when considering systemic medications.

As such, the inclusion of older adults in clinical trials is crucial to better understand how immunosenescence may impact treatment response, or as in our patient's case, how aging may play a role in adverse drug reactions, such as dupilumab-associated psoriasis eruption. Currently, there are limited reports of cases that document this phenomenon. Ogawa et al. described a case of a 71-year-old patient with prurigo nodularis treated with dupilumab injections every two weeks for three months before developing multiple scaly erythematous plaques on the trunk and extremities, consistent with drug-associated psoriatic eruption [12]. In a prospective study by Zhou et al. on the safety of dupilumab in older patients, one 80-year-old patient was reported to have psoriatic eruption after one week of dupilumab treatment [13]. These cases are significant as novel treatments continue to become available in the management of these conditions.

## Conclusions

Although psoriasis continues to be a rare adverse effect of dupilumab, which has a well-documented favorable safety profile, it is important to recognize the presentation of paradoxical psoriatic lesions and to promptly discontinue treatment. Given the gap in the literature documenting dupilumab-associated psoriasis in elderly patients, this case is also valuable, as clinicians consider systemic medications, such as dupilumab, for older adults with refractory dermatitis. Especially since older patients are often concurrently taking several other medications, it is important to recognize rare cutaneous reactions and understand the pathophysiology to know which targeted medications are appropriate.
